# History of DNA Helicases

**DOI:** 10.3390/genes11030255

**Published:** 2020-02-27

**Authors:** Robert M. Brosh, Steven W. Matson

**Affiliations:** 1 Section on DNA Helicases, Laboratory of Molecular Gerontology, National Institute on Aging, National Institutes of Health, Baltimore, MD 21224, USA; 2 Department of Biology, University of North Carolina at Chapel Hill, Chapel Hill, NC 27599, USA

**Keywords:** helicase, DNA replication, DNA repair, recombination, transcription, nucleic acid metabolism, molecular biology, human disease, genomic instability, science education

## Abstract

Since the discovery of the DNA double helix, there has been a fascination in understanding the molecular mechanisms and cellular processes that account for: (i) the transmission of genetic information from one generation to the next and (ii) the remarkable stability of the genome. Nucleic acid biologists have endeavored to unravel the mysteries of DNA not only to understand the processes of DNA replication, repair, recombination, and transcription but to also characterize the underlying basis of genetic diseases characterized by chromosomal instability. Perhaps unexpectedly at first, DNA helicases have arisen as a key class of enzymes to study in this latter capacity. From the first discovery of ATP-dependent DNA unwinding enzymes in the mid 1970’s to the burgeoning of helicase-dependent pathways found to be prevalent in all kingdoms of life, the story of scientific discovery in helicase research is rich and informative. Over four decades after their discovery, we take this opportunity to provide a history of DNA helicases. No doubt, many chapters are left to be written. Nonetheless, at this juncture we are privileged to share our perspective on the DNA helicase field – where it has been, its current state, and where it is headed.

## 1. Discovery of DNA Unwinding Enzymes and Coining the Term Helicase

**Figure 1:**
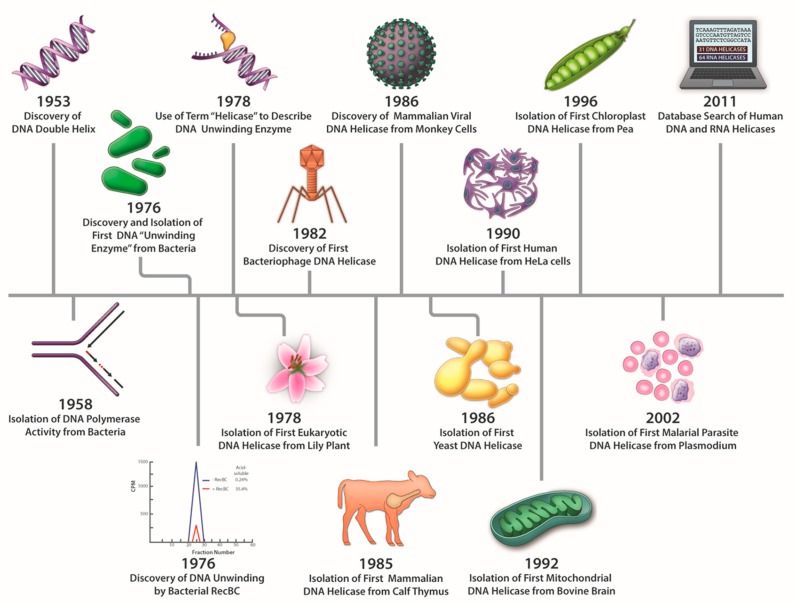
Timeline for discovery of prokaryotic, eukaryotic, and viral DNA helicases. See references and text for details. 1953, Discovery of DNA double helix [16]; 1958, Discovery of DNA polymerase I [17,18]; 1976, Discovery of first bacterial DNA unwinding enzyme [1,2,3]; 1978, “Helicase” term coined [6,7]; 1978, First eukaryotic DNA helicase [19]; 1982, First bacteriophage helicase [20]; 1985, First mammalian DNA helicase [21]; 1986, First mammalian viral DNA helicase [22]; 1986, First yeast DNA helicase [23]; 1990, First human DNA helicase [11]; 1992, First mitochondrial DNA helicase [12]; 1996, First chloroplast DNA helicase [13]; 2002, First malaria DNA helicase [24]; 2011, Genome-wide prediction of human DNA and RNA helicases [11].

The discovery of proteins capable of ATP-dependent enzymatic unwinding of duplex DNA was first reported in 1976 by Hoffmann-Berling and colleagues at the University of Heidelberg [1,2] and Mackay and Linn at the University of California, Berkeley [3]. As pointed out recently in a review by Lohman and Fazio [4], the term “helicase”, referring to an ATP-dependent duplex DNA unwinding enzyme, was coined by Hoffmann-Berling in 1978 [5] and appeared in two subsequent publications in 1979 [6,7]. Even at this early stage, a suggestion was made that different helicase enzymes were unique in terms of properties and mechanism of action (e.g., processivity). Mackay and Linn described *E. coli* RecBC as an ATP-dependent unwinding enzyme in which catalytic duplex strand separation was detectable (under conditions where the nuclease is suppressed by a DNA binding protein), leading them to hypothesize that “the major contribution of RecBC enzyme to recombination would be the unwinding of DNA” [3]. Thus, from both biochemical and genetic viewpoints, the discovery of DNA helicases immediately provoked speculation regarding their mechanisms and biological roles. A timeline for the discovery of prokaryotic, eukaryotic, and viral DNA helicases is shown in Figure 1. Bacteriophage and plant DNA helicases were isolated not long after the discovery of the first bacterial helicases but it would be approximately 10 years until mammalian or yeast DNA helicases were reported. DNA helicase activities from mouse cell extracts were reported in several studies in the late 1980’s and early 1990’s [8,9,10]. The Falaschi laboratory at the International Centre for Genetic Engineering and Biotechnology (Trieste, Italy) isolated the first DNA helicase from human cells in 1990 [11]. Subsequently, DNA helicases were isolated from bovine mitochondria [12] and pea chloroplasts [13], indicating their ubiquitous presence. As of 2011, there were an estimated 95 helicases or putative helicases encoded by the human genome; 31 DNA helicases and 64 RNA helicases [14]. Molecular and cellular studies have elucidated the functional aspects of numerous DNA helicases in various pathways of nucleic acid metabolism. Fifteen years ago, Narendra and Renu Tuteja provided a historical account of prokaryotic and eukaryotic DNA helicases in an informative review [15]. Here, we will highlight some of the major advances in understanding helicase mechanism and function over the years as marked by continued progress in the field.

## 2. Helicase Superfamilies Defined by Sequence Homology and Structure

In 1982, Walker et al. of The MRC Centre (Cambridge, UK) described, from their sequence analysis of α- and β-subunits of ATP synthase, two clustered amino acid regions which shared strong similarity with other ATP-binding proteins [25]. These proteins included a known bacterial ATPase, the recombination protein RecA, adenylate kinase, and myosin from both nematode and rabbit. This led them to propose a common nucleoside triphosphate (NTP) binding fold present in each of these proteins. Six years later, in May of 1988, two reports appeared in *Nature* bringing helicases into the picture. In the first, Gorbalenya and colleagues described an alignment of the amino acid sequence corresponding to the common nucleotide binding fold (proposed by Walker et al., 1982) present in the bacterial RecD protein (a helicase subunit of the RecBCD DNA helicase complex) with a viral NTP motif-containing RNA helicase [26]. In the same issue of *Nature*, Hodgman commented on the presence of seven conserved amino acid motifs found in a superfamily (SF) of proteins implicated in DNA replication and recombination, including the *E. coli* DNA helicases UvrD, Rep, RecB, and RecD [27]. Shortly after, Lane added eukaryotic translation initiation factor eIF-4A, known to be a RNA helicase, and the putative helicase p68 to the classification [28], suggesting an even larger family of helicases or helicase-like proteins. Based on these early visual and computer-aided alignments, the helicase SF consisted of proteins which shared seven distinct highly conserved amino acid segments, or helicase motifs, comprising the two sites (designated A and B) for NTP-binding as they came to be commonly referred [29]. Further sequence alignment revealed a more refined classification of two related SFs with different sets of helicases or putative helicases [30]. To date, six SFs of helicases, putative helicases, or DNA translocases (which lack the classic NTP-driven strand separation activity typical of helicase) have been defined with discovery of new genes [31], and are nicely discussed in a review by Singleton et al. [32].

Site-directed mutagenesis has proven to be a powerful approach to interrogate the biochemical and genetic importance of conserved residues within the helicase motifs. One of the earliest such studies from the Prakash laboratory demonstrated that mutation of the invariant lysine within motif I (ATPase A site) of the *S. cerevisiae* Rad3 helicase abolished its ATPase and unwinding activities, and rendered it inactive in a key post-incision step of nucleotide excision repair (NER) necessary for the removal of ultraviolet (UV) light-induced pyrimidine dimers [33]. Similar mutations in the ATPase A site were found to catalytically inactivate the bacterial PriA [34] and UvrD [35] helicases as well; however, the biological consequences were distinct. Characterization of site-specific mutations in motifs other than the classic ATPase A and B sites have been informative. For example, experimental evidence from the Matson, Wigley, and Keck laboratories demonstrated that motif III serves an important function in coupling DNA binding with ATP binding/hydrolysis and strand separation that is necessary for the efficient catalytic cycles of UvrD [36], PcrA [37], and RecQ [38] helicases, respectively. For further reading about helicase motif structure-function studies and structural perspectives, see [39,40,41].

## 3. Biochemical Studies of DNA Helicases and Their Substrate Specificity

**Figure 2:**
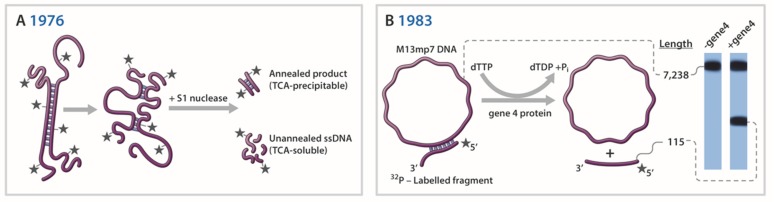

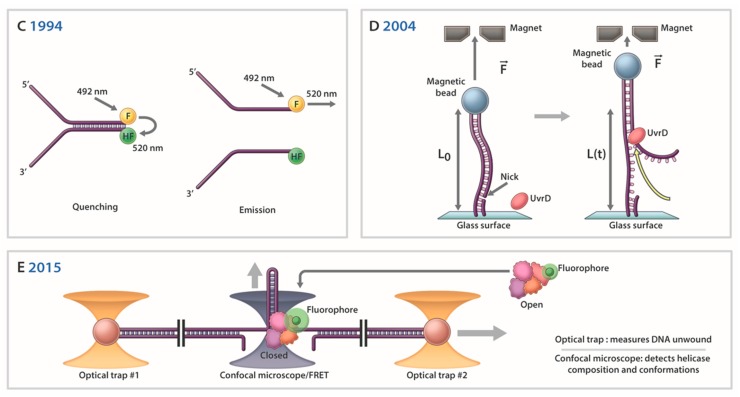
Representative assays to measure helicase-catalyzed DNA unwinding. Dates of published papers using the depicted techniques are indicated in blue font. (**A**) S1 nuclease digestion assay to measure DNA helicase activity. In the example shown, products from helicase reactions containing duplex [^32^P] DNA are incubated with single-strand-specific S1 nuclease and precipitated with cold trichloroacetic acid on membrane filter discs [1]. Total acid-precipitated [^32^P] label was determined by scintillation counting to indirectly measure DNA unwinding. Note: The cartoon schematic depicts small, acid-soluble pieces of ssDNA. (**B**) Native polyacrylamide gel resolution of helicase reaction products. In the example shown, an M13 partial duplex substrate with an annealed [^32^P] DNA fragment (115-mer) was incubated with dTTP and purified bacteriophage T7 gene 4 helicase protein [42]. The helicase reaction products were resolved on a nondenaturing 8% polyacrylamide gel, followed by autoradiography of the wet gel. Scintillation counting of excised radioactive gel slices corresponding to the DNA substrate and unwound ssDNA product would be performed to quantitate helicase activity. A similar strand displacement assay to measure helicase activity in vitro was developed by Nancy Nossal’s laboratory [20] (see text). Interestingly, during the authors’ time together in the Matson laboratory when Brosh was doing research for his PhD thesis at the University of North Carolina at Chapel Hill (1991–1996), PhosphorImager technology largely replaced film autoradiography and scintillation counting for visualization and quantitative analysis of helicase activity measured in vitro. (**C**) Fluorescence resonance energy transfer to monitor helicase-catalyzed DNA unwinding continuously. In the example shown, upon separation of the two complementary strands fluorescence emission from fluorescein (F) excitation can be monitored by a photosensor because it is not quenched by the hexachlorofluorescein (HF) [43]. Data can be collected in real-time by fluorescence instrumentation equipped with a stopped-flow device. (**D**) Single-molecule detection of helicase-catalyzed DNA unwinding using optical tweezers. In the example shown, DNA unwinding is detected by the movement of the magnetic bead subjected to a force (F) induced by a magnetic field gradient [44]. Helicase-catalyzed DNA unwinding results in a stretched single-strand (molecule extension (L)) which enables the magnetic bead to migrate toward the external magnet away from the immobilized DNA end tethered to the coverslip. Video-tracking is employed to detect and measure migration of the microsphere. (**E**) Combined dual-trap optical tweezers and SM fluorescence microscopy to simultaneously detect and measure unwinding activity and conformation of a DNA helicase protein site-specifically labeled with a fluorophore(s) [45]. The figure depicts an experimental system with the two indicated microspheres in dual optical traps tethered by a DNA hairpin. The confocal microscope (green) detects the conformation of the fluorescently labeled helicase protein complex actively unwinding the duplex DNA hairpin. Reference [46] provides a detailed protocol describing this technique dubbed high-resolution “fleezers”.

Over the years, remarkable progress has been made in developing experimental techniques to detect and measure helicase-catalyzed DNA unwinding. Figure 2 shows representative helicase assays illustrating different biochemical and biophysical strategies that have been utilized over time. Of course, there are many applications and experimental techniques for studying DNA helicases which we are unable to address due to space limitations and the enormity of the field which continues to grow. In the immediate sections we will describe some fundamental lessons learned about the properties and mechanisms of DNA helicases using novel experimental procedures and strategies. A special section found later in the text highlights single-molecule (SM) approaches that are gaining in popularity to measure helicase activity.

### 3.1. Helicase Directionality

Early studies of DNA helicase enzymes focused on their single-stranded DNA (ssDNA) stimulated ATPase activity in addition to their helicase activity [1,2,6,7]. This, together with the apparent requirement of ssDNA for a helicase to catalyze duplex DNA unwinding, suggested an interaction with ssDNA leading to the notion of directionality of DNA translocation and unwinding. By its very nature, the DNA double helix is defined by two DNA strands with opposite chemical polarities wrapped around each other in a helical fashion and interacting through a series of noncovalent hydrogen bonds between the two strands [16]. The polarities of the two DNA strands are determined by the sugar-phosphate backbone in which the oxygen atom of the phosphodiester bond is covalently bonded to either the 3’ or 5’ positions of the intervening ribose moiety. The two strands of the DNA double helix run in opposite directions, dictating its anti-parallel nature. This structural feature of the DNA double helix has profound implications for its metabolism, including the copying of the genetic information by the process of semi-conservative DNA replication.

Unidirectional translocation by a helicase along ssDNA, fueled by the energy of nucleoside triphosphate hydrolysis, would in principle provide a mechanism whereby the directionality of duplex DNA unwinding would be established. Indeed, several laboratories established the directionality for the bacteriophage helicases T7 gene 4 protein and T4 gene 41 protein, as well as the bacterial Rep helicase (reviewed in [47]). In a seminal study by Yarranton and Gefter in 1979 radiolabeled partial duplex DNA substrates with defined polarities were incubated with purified Rep protein and ATP followed by ssDNA-specific S1 nuclease digestion (see Figure 2A) and DNA precipitation by trichloroacetic acid. Acid insoluble radioactivity was quantified to determine the fraction of duplex DNA susceptible to nuclease and to infer if a partial duplex substrate with a 3’ or 5’ single-stranded tail was unwound. These experiments indicated that Rep translocates 3’ to 5’ along the single-stranded region of the substrate to reach and unwind the adjacent duplex [48].

Although assays which relied on nuclease sensitivity or isolation of products by sucrose gradient or bound to nitrocellulose were useful tests for helicase-catalyzed DNA unwinding [1], the choice of DNA substrates was narrow and experimental techniques were tedious and relied on indirect measurements. For improved quantitation and direct visualization of helicase reaction products, a more straightforward assay was in demand. The first direct demonstration that a DNA helicase employs its unidirectional translocation on ssDNA to unwind duplex DNA in a directionally specific manner was provided by Nancy Nossal’s laboratory at the NIH in 1982 [20]. A radiolabeled partial duplex DNA substrate characterized by two different length duplex DNA fragments separated by an intervening long ssDNA tract was incubated with the T4 gene 41 protein in the presence of ATP and the reaction products were resolved on a non-denaturing polyacrylamide gel. Using this technique, it was demonstrated that T4 gene 41 moves in a 5’ to 3’ direction with respect to the strand on which it is bound. In 1983 Matson et al. described a similar DNA substrate in which a complementary strand of a radioactively labeled denatured DNA fragment of defined size was annealed to circular single-stranded M13 viral DNA (Figure 2B). Here the substrate had an additional feature in that it possessed a noncomplementary 3’-tail, thereby resembling a forked duplex [42]. This DNA substrate, and its derivatives in which the length of the noncomplementary single-stranded tail was varied, enabled the authors to determine the minimal DNA substrate requirements of T7 gene 4 protein to unwind the DNA duplex adjacent to the junction. The advantage of a direct helicase assay to measure DNA unwinding was convincingly evidenced by the demonstration that the bacterial UvrD helicase, an enzyme with a vital role in both NER and mismatch repair (MMR), translocates 3’ to 5’ in its unwinding mechanism [49], opposite to the directionality of T4 gene 41 or T7 gene 4 helicase proteins. As discussed by Matson in that paper, the 3’ to 5’ directionality of UvrD helicase has implications for the mechanisms of both the MMR and NER pathways.

The mechanism(s) whereby a given DNA helicase achieves unidirectional polarity of movement on DNA gained considerable interest in the late 1990’s. Some insight into the mechanism of helicase DNA translocation and directionality was garnered from structural and biochemical studies. Relative DNA binding affinity of the two RecA motor domains and protein contacts with ssDNA of a defined polarity are likely to dictate directionality of helicase movement coupled to ATP hydrolysis, as suggested by structural studies of the *B. stearothermophilus* PcrA helicase [50,51] and the *E. coli* Rep helicase [52]. The precise mechanistic details are likely to be different depending on the helicase.

In 2009, the Wigley lab published a paper that provided a mechanistic basis for how the sequence-related *D. radiodurans* DNA helicase RecD2 translocates on DNA with an opposite polarity (5’ to 3’) to that of PcrA. Their studies suggested that relative grip strength of defined protein domains in the helicase dictate its directionality of movement as opposed to opposite orientations of ssDNA binding sites [53]. This theme resurfaced in research on the XPD helicase from the Kisker and Spies laboratories who published papers in 2012 which shed light on its 5’ to 3’ directionality of translocation. The co-crystal structure of *T. acidophilum* XPD with a single-stranded oligonucleotide showed the trail of the translocated strand as it traversed the helicase pore; a comparison of the XPD structure with structures of DNA helicases that translocate with the opposite polarity (3’ to 5’) indicated the same orientation of DNA binding by the two RecA domains, suggesting that a reversal of protein gripping is responsible for the different polarities [54]. Studies that mapped XPD protein contacts on DNA demonstrated that *T. acidophilum* XPD binds ssDNA with an orientation similar to DNA helicases that translocate 3’ to 5’; one of the RecA domains in collaboration with the iron-sulfur (Fe-S) domain most likely provides the wedge for duplex separation [55].

### 3.2. Helicases Resolve Unconventional DNA Structures

In early experiments helicase-catalyzed DNA unwinding was assessed using partial duplex DNA substrates with a single-strand tail adjacent to the duplex region to allow helicase loading. However, these simplistic double-stranded DNA (dsDNA) molecules do not reflect the in vivo situation. An example, noted above, is the T7 gene 4 protein which requires a forked duplex DNA molecule [42]. Moreover, nucleic acid metabolic processes that occur in cells are relevant. During transcription stable DNA:RNA hybrids may form, causing one of the strands of the original DNA duplex to be displaced. These so-called R-loops may potentially interfere with the progress of an advancing replisome, suggesting that their timely resolution is important for genomic stability [56]. A role for a DNA helicase in this capacity was first suggested by experimental evidence that UvrD unwinds stable DNA:RNA hybrids even more efficiently than DNA duplexes in vitro [57]. The poor ability of RNA to serve as an effector for UvrD-catalyzed ATP hydrolysis [58] suggests a DNA-specific loading and interaction mechanism for unwinding the DNA:RNA hybrid. Supporting this general principle, to our knowledge the vast majority of DNA helicases characterized to date which bind DNA with higher affinity than RNA also preferentially unwind DNA duplex substrates compared to RNA duplex substrates. In addition to UvrD, the replicative bacterial, archaeal, and eukaryotic DNA helicases can unwind RNA-DNA substrates [59].

Over time, researchers investigated the activity of select helicases on nucleic acid structures that arise in specific pathways of cellular DNA metabolism. One such substrate is the Holliday Junction (HJ), a branched DNA molecule with four double-stranded arms that represents a key intermediate of genetic recombination and double-strand break repair that occurs during the S/G2 phases of the cell cycle. Building on genetic studies from Bob Lloyd’s laboratory demonstrating the importance of *E. coli* RuvA, RuvB, and RuvC in recombinational repair [60] and the finding that RuvC interacts with HJs to catalyze their symmetrical endonucleolytic cleavage [61], Steve West’s laboratory determined that the ATPase/helicase RuvB together with the DNA binding protein RuvA catalyzed HJ branch-migration in an ATP-dependent manner [62]. Thus, the RuvA/B helicase complex was determined to be capable of interacting with and branch-migrating a HJ structure that lacked any extensive ssDNA character. This discovery was significant and set a precedent for later work showing that human RecQ helicases involved in homologous recombination (HR) repair and implicated in diseases of premature aging and cancer (e.g., BLM [63], WRN [64]) also bind tightly to and branch-migrate HJs in an ATP-dependent manner [65] (see *Genetic Diseases*, Section 10). It should be noted that UvrD helicase also binds and unwinds HJ DNA substrates [66] likely explaining (at least partially) the recombination phenotype associated with *uvrD* mutants.

Although it is generally true that a given DNA helicase requires a ssDNA loading tail to catalyze unwinding of the adjacent duplex DNA (at least in vitro), there are exceptions. At relatively high helicase protein concentrations, *E. coli* RecQ was reported to unwind blunt duplex DNA substrates [67]. Thermal breathing or fraying of the duplex end promoted by helicase binding to the DNA substrate may play a role in this reaction. UvrD helicase, at elevated protein concentrations, was also found to initiate unwinding of duplex DNA from blunt ends as well as nicks [68]. The latter may provide an entry site for UvrD during DNA MMR [69,70] or NER [69,71]. Although a role for UvrD in post-incision strand release in bacterial MMR was elucidated in the early 1990s, we still do not know the identity of the helicase(s) (if any) implicated in this strand release step of eukaryotic MMR.

**Figure 3:**
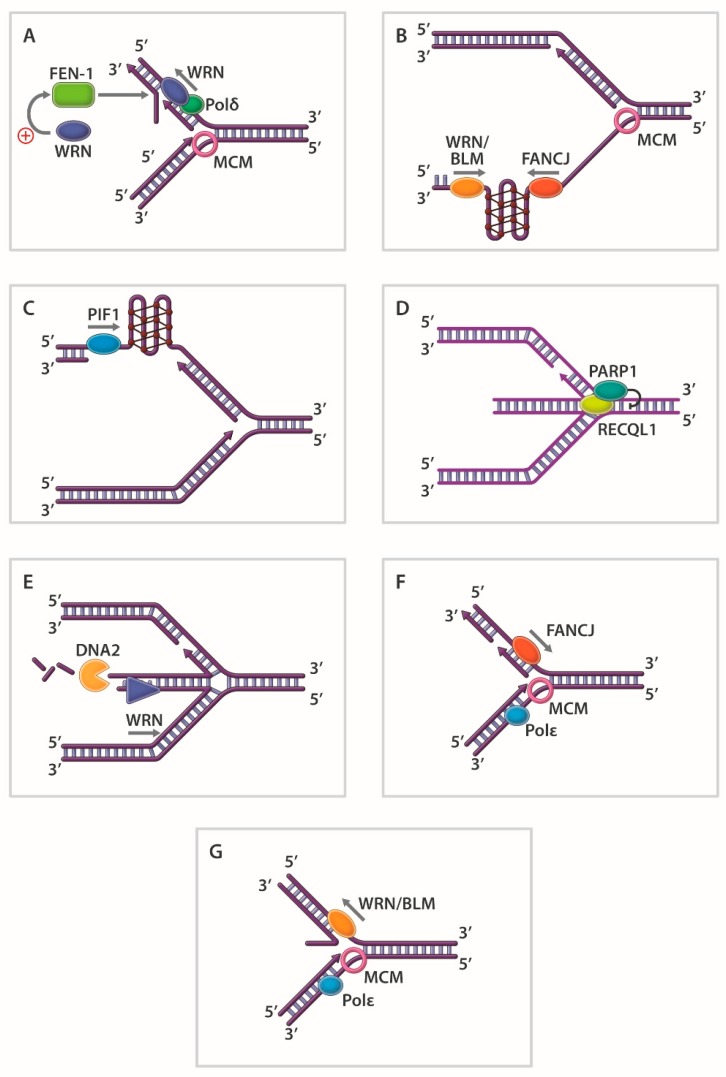
Replication fork structures acted upon by DNA helicases. (**A**) WRN [72] (or BLM [73,74]) unwind 5’ flaps thereby promoting strand displacement DNA synthesis. In addition, WRN [99,100,101] or BLM [74,102] interacts with FEN-1 and stimulates its 5’ flap endonuclease activity to aid in Okazaki fragment processing. (**B**) WRN or BLM collaborates with FANCJ to resolve G4 structures that impede DNA synthesis [84]. (**C**) Pif1 resolves G4 structures on the lagging strand template to allow smooth DNA synthesis [97]. Note: this depiction does not preclude a role of Pif1 in certain contexts to resolve G4 in the leading strand template. (**D**) RECQL1 reverse branch-migrates regressed fork to restore the replication fork in a manner that is negatively affected by PARP1 [103]. (**E**) DNA2 nucleolytically processes reversed forks in a process aided by WRN helicase to promote fork restart [104]. (**F**) The replication fork stalls due to DNA damage or replication stress. FANCJ promotes fork elongation by a mechanism not fully characterized. The fork remodeling factor (not shown) may oppose FANCJ’s role in elongation [105]. (**G**) *E. coli* RecG [106] or human WRN [107,108]/BLM [107,109] unwind lagging strand duplex to initiate fork regression when fork stalls. Note: For simplicity, not all fork-interacting proteins are shown in panels. Also, these panels are meant to be representative of DNA structures found at the fork. Not all DNA structures or helicases are shown.

In other work, it was observed that the human RecQ 3’ to 5’ DNA helicases WRN [72], BLM [73,74], and RECQL1 [75] efficiently unwind 5’ flap DNA substrates despite the absence of a pre-existing 3’-ssDNA tail (Figure 3A). These studies, supported by mechanistic experiments with WRN helicase [72], suggest that junction-specific DNA binding and strand-specific loading are important properties of certain DNA helicases. The 5’ flap substrate is an important intermediate during Okazaki fragment maturation or strand displacement DNA synthesis in base excision repair. The coordinate action of human RecQ helicases with structure-specific Rad2 nucleases (FEN-1, EXO1) on 5’ flap DNA substrates is detailed in a number of studies (reviewed in [76]) (Figure 3A). Helicases also act upon unusual DNA structures that deviate from the canonical DNA double helix. DNA triplexes, G-quadruplexes (G4), and left-handed Z-DNA are prone to form at specific DNA sequences [77]. Such alternatively arranged DNA conformations potentially influence replication and transcription. In addition, these alternate DNA structures are proposed to form at specialized regions of chromosomal DNA (e.g., G4: guanine-rich telomeres, promoter elements, ribosomal DNA; triplex DNA: polypurine/polypyrimdine tracts; Z-DNA: alternating G/C sequence elements) and have consequences for genomic stability. Over the years, a number of groups have obtained biochemical evidence that DNA helicases resolve triplex DNA and G-quadruplex DNA in vitro (see Table 1 for representative examples).

Although beyond the scope of this review, biological and molecular evidence suggest that certain DNA helicases preserve genomic stability and maintain cellular homeostasis by resolving such alternate DNA structures. G4-resolving helicases are discussed in recent reviews [78,79]. A seminal study from the Lansdorp laboratory first suggested that a putative DNA helicase known as DOG-1 in *C. elegans* suppressed the accumulation of G4 structures in the lagging strand ssDNA template thereby preventing deletions in regions of the genome characterized by guanine-rich DNA [80]. Later studies substantiated important and conserved roles of DOG-1 homologs (FANCJ) in G4 DNA metabolism [81,82,83] (Figure 3B), including epigenetic regulation [84,85]. For an interesting perspective on FANCJ and the G4-resolving helicases RTEL1 and BLM, readers are encouraged to read reference [86].

Evidence suggests that Pif1 family helicases from species ranging from bacteria to human [87] resolve G4 DNA structures in vitro [88,89,90,91,92,93,94,95]. A role of PIF1 in G4 DNA metabolism in vivo is perhaps best exemplified by *S. cerevisiae* Pif1 [89,90,91,96], the founding member of the Pif1 family of helicases. Recent results from a live-cell imaging approach demonstrated that replication rates are significantly reduced by genomic DNA sequences predicted to form G4 in the lagging strand template but not the leading strand template in individual yeast cells [97]. The stimulatory effect of Pif1 on DNA replication was dependent on its interaction with PCNA, suggesting that Pif1 G4 resolution coupled with progression of the replication machinery is crucial for efficient DNA synthesis of the lagging strand (Figure 3C).

As elaborated upon below in Section 9.2, experimental evidence suggests that the XPB and XPD helicases are involved in transcriptional regulation by their interactions with G4 DNA structures associated with gene promoters [98].

## 4. Proposed Models for Helicase-Catalyzed DNA Unwinding

As it became apparent in the late 1970s and early 1980s that there are numerous DNA helicases with defined biochemical properties and DNA substrate specificities, an immense interest in characterizing their mechanisms of action developed. This led to more refined studies of helicase proteins, incorporating biochemical and biophysical approaches. Not only were researchers investigating protein assembly states but also changes in the conformation and functionality of the helicase that were induced by NTP and DNA binding. Efforts in these areas, along with new information on the structures of DNA helicase proteins, fueled even greater attention to mechanisms for DNA unwinding. In the following sections, we will discuss some prominent models for helicase-catalyzed DNA unwinding.

### 4.1. Rolling Model for Rep Helicase-Catalyzed Duplex DNA Unwinding

Significant advances in mechanistic studies of DNA helicases kicked into high gear in the late 1980s and early 1990s with the availability of abundant quantities of highly purified homogenous recombinant helicase protein that had been overexpressed in bacteria [125]. Research from Tim Lohman’s laboratory initially focused on the molecular mechanism of *E. coli* Rep helicase. Using biophysical and biochemical techniques, the Rep assembly state and the effects of nucleotide binding on Rep’s interaction with ssDNA or dsDNA were examined. Based on their studies, Wong and Lohman proposed a rolling model for Rep-catalyzed duplex DNA unwinding in which allosteric effects of nucleotide binding influenced the protein’s interaction with ssDNA or duplex DNA thereby providing a means for Rep to be continuously bound to the DNA substrate it was actively unwinding [126]. The data leading to the proposal of the rolling model is also consistent with the inch-worm model (see below), commonly accepted today. This advance for understanding helicase mechanism suggested that conformational changes (induced by nucleotide and DNA binding [127]) would affect oligomerization state, unwinding activity, and helicase processivity. A series of papers detailing kinetic analyses of Rep helicase suggested the functional cooperativity of Rep monomers, supporting a dimeric mode of action for Rep helicase that may be applicable to other DNA helicases (for review, see [128]). For example, the dimeric form of the FANCJ helicase (implicated in Fanconi Anemia (FA) see *Genetic Diseases* section, Section 10) was found to have a greater specific activity for ATPase, DNA binding, and helicase, compared to the monomer [129]. Further studies of FANCJ oligomerization should address the role of ligand-induced conformational changes in its mechanism of unwinding. Moreover, a structural determination of FANCJ, as well as the sequence-related DNA helicases RTEL1 and DDX11, and their interactions with ssDNA and dsDNA will be helpful.

### 4.2. Inchworm Model for PcrA Helicase-Catalyzed Duplex DNA Unwinding

In contrast to the evidence for Rep dimerization, the sequence-related PcrA helicase from *B. stearothermophilus* was determined by Bird et al. at the University of Oxford to exist solely as a monomer in 1998 [130]. This observation led the authors to propose that PcrA’s mechanism of DNA unwinding was distinct from that of a rolling model involving two (or more) subunits. Harkening back to an earlier related model of helicase action proposed by Yarranton and Gefter [48], the ability of PcrA to interact simultaneously with both ssDNA and dsDNA and additional evidence prompted Dale Wigley’s laboratory to propose an inchworm model for PcrA unwinding in which the monomeric helicase extends into duplex DNA as it translocates in an ATP-dependent fashion [130]. The step size for duplex unwinding is inferred to be as short as 1 base pair (bp) for an inchworm helicase, whereas a helicase that operates by a rolling model may have a longer step size reflecting the DNA binding site size for each of the monomers.

Subsequent structural work from the Wigley laboratory demonstrated that the PcrA helicase monomer is capable of binding ssDNA and duplex DNA simultaneously when ATP, modeled by the ATP analog ADPNP, is bound to the enzyme [51]. In this mode, PcrA assumes a conformation in which the relative positions of the two RecA domains favor close interaction of the protein surface to duplex DNA. When ATP is hydrolyzed, modeled by a bound sulfate ion, the two RecA domains of PcrA assume a rigid conformation that reflects a conformation of the helicase protein complexed solely with ssDNA. Thus, a conformational change in PcrA induced by intrinsic ATP hydrolysis causes the helicase protein to dissociate from duplex DNA but remain bound to ssDNA. Consecutive cycles of DNA translocation and destabilization of duplex patches as short as 1 bp controlled by conformational changes in PcrA elicited by nucleotide binding and hydrolysis constitute the basic actions of the inchworm mechanism.

Interestingly, seven years later the Yang laboratory proposed a combined wrench-and-inchworm mechanism for unwinding by the sequence-related UvrD helicase in which binding of a single ATP molecule results in 1 bp of duplex DNA unwound and the subsequent release of ADP and P_i_ allows translocation of UvrD on the emerging single-strand [131].

### 4.3. Helicase Processivity, Functional Cooperativity, and Protein Displacement

Although the assembly state of helicases has been of considerable interest over the years, functional cooperativity of multiple helicase molecules in unwinding a given duplex DNA substrate may involve elements other than protein oligomerization. For example, UvrD helicase unwinds partial duplex DNA substrates of increasing duplex lengths by a protein concentration dependent mechanism in which the number of bp unwound is directly proportional to the helicase concentration in the reaction mixture [58]. Although not formally tested, a model was proposed suggesting that UvrD helicase molecules concentrate at the ssDNA: dsDNA junction, allowing unwinding of longer duplex lengths in a non-processive fashion. The limited helicase processivity of UvrD may be tailored for its functions in NER or MMR, which require the unwinding of relatively short duplex DNA tracts as compared with unwinding at the replication fork in which many thousands of bp are typically encountered by the bacterial replicative helicase DnaB (see *Replicative Helicase* section below). Not all helicases lack processivity. For example, the TraI helicase, which functions in conjugative DNA transfer, is a highly processive helicase that functions as a monomer to unwind duplex DNA tracts greater than 850 bp [132].

Kevin Raney’s laboratory has a long-standing interest in the requirements for optimal unwinding of duplex DNA molecules by helicase proteins. Using pre-steady state kinetic measurements, they determined that the hepatitis C virus helicase NS3 unwinds long DNA tracts by aligning multiple NS3 molecules on the ssDNA on which it translocates, visualized conceptually by an array of locomotives advancing together on a train track [133]. In contrast to this model for functional cooperativity among NS3 helicase molecules, experimental evidence suggests that trailing Dda helicase molecules (from phage T4) do not exert a significant effect on the leading Dda monomer for unwinding conventional duplex DNA substrates [134]. Therefore, unique differences in the train helicase model are likely to exist.

Roadblocks to helicase translocation, such as protein bound to the DNA, may evoke unique mechanisms of helicase action as it was reported that trailing Dda helicase molecules contacting the lead Dda molecule serve to facilitate protein displacement [135]. This functional cooperativity among Dda helicase molecules to displace protein bound to DNA is a mechanism that potentially contributes to the ability of Dda to dissociate a stationary RNA polymerase and allow a faster rate of fork movement by the T4 bacteriophage replication machinery, as originally shown in a landmark 1983 paper by Bedinger et al. [136]. The demonstration that Lac repressor protein bound tightly to the double-stranded region of a partial duplex DNA substrate exerts differential effects on the ability of various DNA helicases to displace it and unwind the underlying duplex suggests that certain helicases may have specialized roles in clearing protein roadblocks to DNA replication [137]. The McGlynn laboratory has made considerable progress in this area, providing evidence that accessory bacterial DNA helicases (e.g., Rep, UvrD) can enable the replicative DnaB helicase to bypass protein obstacles assuring smooth replisome progression [138]. For a review on this topic, see [139]. Biochemical studies demonstrate that the eukaryotic ssDNA binding protein RPA, which physically interacts with several SF2 RecQ and Fe-S helicases and increases their duplex DNA unwinding activity [140,141,142,143], stimulates helicase-catalyzed protein displacement [144]. Certain DNA helicases (e.g., yeast Srs2) displace the major strand exchange protein Rad51 bound to DNA [145,146], a function that is important to manage or control HR.

### 4.4. Strand Exclusion Model for Replicative Helicases with Multimeric Ring-Like Structures

The prototypical replicative helicases are represented by the bacterial DnaB and eukaryotic minichromosome maintenance (MCM) helicases, which each form a hexameric ring-like structure of the same (DnaB) or different (Mcm2–7) subunits (for review, see [147]). Both DnaB and MCM helicases are believed to unwind dsDNA by inserting one strand of the unwound duplex inside the donut ring and the other outside the ring. This mechanism is classically known as steric exclusion. However, recent studies suggest that ring-like helicases are characterized by subtle unique properties in terms of their interactions with the “excluded” strand [148]. Interestingly, the bacterial DnaB helicase operates with a 5’ to 3’ directionality placing it on the lagging strand template whereas eukaryotic MCM translocates 3’ to 5’ on the leading strand template. It should be noted that replicative helicases can translocate dsDNA [59,149].

The discovery of DnaB helicase as an essential factor in *E. coli* chromosomal DNA replication was achieved through genetic analyses from several groups [150,151,152,153,154] before the helicase activity of the protein was demonstrated. These studies, and additional findings from cellular and biochemical analyses of bacteriophage replication, set the stage for two biochemists from The Johns Hopkins University to hypothesize that DnaB was responsible for processive unwinding of duplex DNA at the replication fork. LeBowitz and McMacken biochemically tested purified DnaB for ATP-dependent DNA unwinding activity on a M13 partial duplex DNA substrate with over 1000 bp of duplex DNA and two preformed forks [155]. They subsequently proposed a mechanistic model for the functions of the major replication proteins at an *E. coli* replication fork. Following that, a series of papers from Arthur Kornberg’s laboratory (which began with the finding that in a reconstituted system DnaB helicase function is required for initiation of DNA synthesis at the chromosomal origin [156]) and other laboratories provided a more complete model that served as a gold standard for future studies on the mechanism of eukaryotic DNA replication. For Kornberg’s perspective on the lessons learned during the pursuit of characterizing the mechanism of DNA replication and the enzymes involved, see [157].

As discussed in a comprehensive review by Bochman and Schwacha [158], the *MCM* genes were first discovered in *S. cerevisiae* by the Tye laboratory employing a screen to detect mutants that were defective in the regulation of replication initiation [159]. Subsequent studies in yeast [160,161,162] and *X. laevis* [163,164,165] provided further evidence for their involvement in various stages of cellular DNA replication. Much of the early work on the biochemical characterization of MCM ATPase/helicase function, assembly state, and interaction with nucleic acid was performed with the homologous archaeal proteins [166], which proved to be (and still are) highly informative for the analysis of eukaryotic MCM (see [158] for details). The first demonstration that the MCM protein complex has ATPase and DNA helicase activity came from studies by Yukio Ishimi [167]. The intricate mechanistic functions of the MCM helicase complex continue to be a fascinating area of detailed study offering new insights into its roles in DNA replication licensing, initiation, progression, and termination (for review, see [168,169,170]). Of particular interest in recent years is the mechanism(s) utilized by the MCM helicase complex to handle DNA roadblocks and the functional consequences for obstacle bypass (reviewed in [171]). Recently, a crystal structure of *S. solfataricus* MCM bound to ssDNA, an ATP analog, and ADP was solved which indicated a rotary mechanism to translocate DNA that is conserved for both archaeal and eukaryotic MCM helicases [172]. A model was suggested for the conversion of the MCM double-hexamer encircling dsDNA to single hexamers encircling ssDNA that bears relevance to replication initiation.

## 5. DNA Helicase Protein Structures

Structural information derived from biophysical approaches has provided important insights to mechanistic features of how various DNA helicases unwind duplex DNA. A summary of the major discoveries of prominent DNA helicase structures is shown in Table 2. This summary is not meant to be exhaustive but rather representative for members of the various helicase families and to provide the reader an opportunity to consult the cited references for more in-depth discussion of the findings. The first structural information for a DNA helicase that can be found in the literature is from the Hurwitz laboratory in 1989 in which they reported that the Simian virus 40 (SV40) large tumor (T) antigen, required for initiation of viral replication, forms ATP-dependent double hexamers around dsDNA as revealed by scanning transmission electron microscopy [173]. In 1995, a significant advance was made by the Egelman and Patel laboratories in their analysis of the bacteriophage T7 helicase/primase [174]. They utilized electron microscopy, three-dimensional reconstruction, and protein crosslinking to show that the T7 gene 4 protein forms a hexameric ring that encircles ssDNA. Moreover, they showed the protein is bound to the DNA with a defined polarity, suggesting a structural basis for the 5’ to 3’ directionality of helicase movement on DNA and the mode of unwinding. These and other studies provided the foundation for the field’s current appreciation that replicative helicases, including *E. coli* DnaB [175] and MCM [176,177], form ring-like structures around DNA to enact DNA unwinding. However, the molecular mechanics and functions of DNA unwinding by the replicative helicases (e.g., MCM) is informed by not only structural characterization but also SM and biological studies (for review, see [178]).

In 1996, Dale Wigley’s laboratory reported the first crystal structure of a DNA helicase, which revealed that PcrA helicase has two RecA-like domains that form a cleft for ATP binding [50]. This discovery was closely followed by the determination of the first structure of a DNA helicase (Rep) bound to DNA, which suggested major domain swiveling coupled to DNA translocation [52]. A subsequent study of PcrA bound to DNA in the presence of a nucleotide analog led Wigley’s team to further define the inchworm mechanism for helicase-catalyzed DNA unwinding by SF 1 helicases [51] that remains prevalent today (see Section 4.2). With advances in understanding the molecular architecture of SF1 helicases, the Keck laboratory at the University of Wisconsin Medical School solved the structure of a SF2 helicase, namely *E. coli* RecQ, in 2003 [179]. This work revealed the structures of two auxiliary domains (Zn^2+^ binding; winged helix) in addition to the conserved RecA-like domains implicated in nucleotide binding. The *E. coli* RecQ structure provided insight into the molecular defects caused by missense mutations in a human RecQ helicase linked to the genetic disease Bloom syndrome (BS), discussed below [180,181,182].

Mechanistic features of how the *E. coli* RecBCD helicase-nuclease processes double-strand breaks were elucidated by structural determination of the protein complex [189]. The bipolar helicase complex has the two motor subunits (RecB, RecD) on opposite strands at the fork of the duplex with the RecC subunit positioned to split the two strands. The architecture of the DNA:protein complex enables nucleolytic cutting by RecB in a manner that is orchestrated by RecC’s recognition of the recombinational hotspot (Chi site).

In 2008, three laboratories in different countries (Tainer in the United States; White in the United Kingdom and Kisker in Germany) independently reported the crystal structures of the NER helicase XPD from three different archaeal species, providing evidence for the structural importance of a conserved Fe-S cluster residing within the helicase core domain and acting as a wedge during duplex DNA unwinding [191,192,193]. Steady progress in understanding the structures of RecQ helicases has continued, highlighted by a 2016 paper in which experimental evidence provided by the Gileadi and Vindigni laboratories implicated a strand separation pin buttressed by a protein dimer interface in human RECQL1 to mediate duplex DNA separation [196].

Although the focus of structural studies has historically been to elucidate the molecular mechanism for unwinding dsDNA, in 2018 two papers appeared which addressed the structural elements and molecular details for resolving G-quadruplex DNA by the *S. cerevisiae* Pif1 [201] and *C. sakazakii* RecQ [202] helicases. This area of research is likely to blossom with the growing interest in the roles of unconventionally structured DNA in genome biology.

## 6. Single-Molecule Studies of Helicase-Catalyzed DNA Unwinding

Bulk biochemical measurements of DNA helicase activity have been highly informative for characterizing substrate specificity, modulation of catalytic activity by protein interactions and post-translational modifications, and structure-function studies of site-directed mutant helicase proteins. However, detailed mechanistic studies of helicase function required more sophisticated and sensitive assays. This led researchers to develop experimental strategies to perform SM studies in which the molecular interactions of a helicase protein with its DNA substrate could be determined from a single event as opposed to an average of many events. The first published studies of a DNA helicase using SM strategies were performed with the *E. coli* RecBCD helicase/nuclease. In 2001, two papers appeared in *Nature* describing SM experiments to assess RecBCD’s DNA translocation and unwinding functions [204,205]. The strategies employed in the two studies were different. Bianco et al. visualized single RecBCD molecules as they moved on dsDNA by optically trapping individual fluorescently labeled dsDNA molecules attached to polystyrene beads and monitoring fluorescent dye displacement from the DNA substrate by RecBCD as the DNA substrate was unwound [204]. Their results indicated that RecBCD is highly processive, unwinding duplex lengths of over 40,000 bp at a rate of ~970 bp per second. In the companion paper, Dohoney and Gelles used tethered-particle light microscopy to detect DNA unwinding by RecBCD at a resolution of ~100 bp with a biotin-tagged RecD subunit bound to polystyrene beads [205]. They observed that most individual RecBCD molecules move at a constant rate as they traverse the duplex, which in the study was determined to be greater than 1 kb in length, in a manner that translocation and unwinding are tightly coupled.

As mentioned earlier, many DNA helicases (e.g., UvrD, Rep) lack the processivity exhibited by the RecBCD complex. To investigate the molecular mechanism of DNA unwinding catalyzed by the Rep helicase, Ha et al. used an assay based on fluorescent resonance energy transfer (FRET) with donor and acceptor moieties attached to opposite strands of a duplex DNA substrate, which in turn was bound to a polymer-coated surface [206]. Structural changes to the DNA substrate could be assessed by a specialized microscope, enabling detection of DNA unwinding, helicase stalling, duplex rewinding, and re-initiation of DNA unwinding. This high-powered approach, and other innovative strategies in SM experimentation, ushered in a new era for mechanistic studies of DNA helicases. Some of the important and diverse innovations in SM studies of DNA helicases are discussed below from a historical perspective, but due to the broad scope of the field readers are encouraged to consult some timely reviews [207,208,209].

In 2004 the Croquette laboratory described an experimental strategy to monitor UvrD helicase activity at the SM level using a nicked duplex DNA molecule anchored on one end to a magnetic lead and the other to a glass surface [44] (Figure 2D). With a force applied to stretch the ssDNA molecule, they were able to measure extensions in the DNA attributed to duplex unwinding. Based on their experimental results, the authors suggested a model in which strand switching by UvrD during dsDNA unwinding elongation allows rapid reannealing behind the translocating helicase. Over the years, models that include unwound DNA strands rezipping have become popular, and evidence for this has been derived from varied SM strategies. For example, in 2005 Myong et al. published a paper describing a site-specific modification of the Rep protein which labeled it with a dye (donor) so that helicase movement could be measured by FRET as it translocated away from a site-specific DNA acceptor moiety [210]. Using this approach, they were able to show that Rep monomer undergoes repetitive shuttling on DNA induced by a duplex or protein bound to the DNA. Several nice reviews from prominent laboratories appeared at this time which summarized insights into helicase mechanisms garnered by SM studies [211,212,213].

A long-standing question in the field centered on whether the ring-like replicative helicases unwound duplex DNA using a passive or an active mechanism. The passive mechanism of unwinding relies on thermal fluctuations at the fork that make available ssDNA which is then trapped by the replicative helicase as it translocates along the DNA lattice. Alternatively, certain models for helicase-catalyzed duplex DNA unwinding suggested an active unwinding mechanism in which the helicase protein itself destabilized the duplex region at a ssDNA:dsDNA junction. Both mechanisms are dependent on NTP hydrolysis, but the salient features of unwinding are distinct. To address this, Johnson et al. performed SM studies using individual T7 gene 4 protein helicase hexamers with DNA substrates of defined nucleotide sequence subjected to a destabilizing force at the fork junction [214]. Their results were consistent with an active mechanism of DNA unwinding by T7 gene 4 helicase.

The efficient coupling of a replicative DNA polymerase with a helicase has been a topic of considerable interest over the years. Dating back to 1996, the Marians laboratory determined that a protein interaction between the *E. coli* DNA polymerase III holoenzyme and the DnaB replicative helicase is responsible for rapid replication fork movement [215]. In 2005, the Patel laboratory provided evidence for a mechanical coupling between the T7 DNA polymerase and helicase, demonstrating that DNA synthesis accelerates helicase-catalyzed DNA unwinding [216]. Consistent with this work, in 2007 Charles Richardson’s laboratory reported that T7 DNA polymerase and gene 4 helicase protein interact to form a highly processive replication fork complex [217].

Structural characterization of RecBCD by the Wigley lab [189], discussed above, helped to elucidate the mechanism of the helicase-nuclease complex. The two motor subunits (RecB, RecD) of the RecBCD complex move with opposite polarities posing the unique question of how their motors are coordinated and what happens when the helicase encounters a recombination hotspot DNA sequence known as Chi. SM analysis suggested a model of lead motor subunit switching to explain RecBCD’s biological roles [218]. In addition to playing a fundamental role in HR, RecBCD also degrades foreign DNA via its intrinsic nuclease activity.

Helicase unwinding rates may be slowed by proteins bound to DNA, as revealed by the Spies’ laboratory SM studies of the XPD helicase [219]. However, certain helicases, like PcrA, are well equipped to strip proteins (RecA filament) bound to DNA [220]. Remarkably, even large protein-DNA adducts cross-linked to the translocating strand can be bypassed by the large T antigen replicative helicase, as demonstrated by ensemble assays [221]. In 2013, Finkelstein and Greene contributed an insightful review detailing SM studies which helped to elucidate how DNA helicases deal with protein obstacles in their path [222]. Intricate details of how the CMG (Cdc45, Mcm2-7, GINS) helicase complex bypasses protein-DNA cross-links to allow their removal by proteolysis are emerging through combined experimental approaches that include SM [223].

Loading of the replicative Mcm2–7 helicase and initiation of DNA unwinding at origins of replication has attracted great interest in the field. Using a SM multi-wavelength fluorescence strategy with distinguishable fluorophores attached to different proteins and FRET-based measurements, Ticau et al. presented evidence in a landmark 2015 paper that bidirectional loading of the Mcm2-7 hexamer complexes is achieved by coordinated and distinct mechanisms to begin origin unwinding and DNA synthesis in opposing directions [224]. Loading of eukaryotic Mcm2-7 helicase complexes to origin-DNA occurs sequentially to form the double-hexamer, a parameter that was not definitively determined in the structural study of *S. solfataricus* MCM by Meagher et al. [172]. Ultimately, the combination of structural, biochemical and kinetic approaches with advanced techniques may provide further elucidation of the precise mechanism of action by MCM during origin unwinding and initiation of DNA synthesis.

Magnetic tweezers-based SM assays have allowed researchers to gain a better molecular understanding of DNA helicases (e.g., RecQ) that act upon specific DNA structures such as the strand invasion intermediate displacement (D)-loop that forms during HR [225]. Various steps in the HR pathway can now be studied at SM resolution with the development of a variety of imaging methods (for review, see [226]). However, measurement of helicase activity under applied force can introduce complexity to the unwinding mechanism that may deviate from physiological conditions. Therefore, studies like a very recent one published by the Galletto laboratory that investigated the DNA unwinding mechanism by Pif1 in real-time using SM Förster resonance energy transfer approaches will continue to be valuable [227].

Less than two decades of research by SM experimentation has provided a wealth of new insights to the molecular mechanisms of DNA helicases. This field has grown tremendously with more sophisticated techniques that have begun to interface with studies of complex DNA processes (e.g., DNA end resection, semi-conservative replication, genetic recombination, DNA repair, transcriptional activation). The modern applications of SM approaches, such as high-resolution “fleezers” [45,46] (Figure 2E), leave little doubt that tremendous advances in our understanding of how helicases mechanistically unwind complex DNA structures and collaborate with other nucleic acid metabolizing proteins are on the horizon.

## 7. Protein Interactions of DNA Helicases

In 1993 Aziz Sancar at the University of North Carolina at Chapel Hill described his molecular matchmaker model as it applied to both NER and MMR in *E. coli* [228]. This description helped pave the way for a whole field of study in protein interactions and their importance in DNA repair, and more broadly in nucleic acid transactions. Identification and characterization of physical and functional interactions between DNA helicases and other nuclear or mitochondrial proteins has accelerated an understanding of cellular and genetic pathways required for genomic stability, a robust DNA damage response, DNA replication, normal gene expression, and ultimately cellular homeostasis. The catalytic efficiency of DNA unwinding by helicases can be modulated by their physical and/or functional interactions with such factors as ssDNA binding proteins (e.g., *E. coli* SSB [229,230,231] or human RPA [140,141,232,233]), shelterin proteins that bind to telomeric DNA sequences (e.g., TRF2 [234]), and DNA polymerases that convert unwound ssDNA to duplex by nascent DNA synthesis [216], as well as many other proteins. Physical interaction of RPA with WRN or BLM helicases plays an important role in stimulation of helicase-catalyzed DNA unwinding [232]. Alternatively, helicases physically bind to nucleic acid metabolizing proteins and alter their functionality. For example, the cleavage efficiency of structure-specific Rad2 nucleases is greatly increased by physical interactions with certain RecQ DNA helicases [76]. T7 gene 4 stimulates DNA synthesis by T7 DNA polymerase [235], a functional interaction that has been found to be relevant for many helicase-DNA polymerase partnerships. The topic of helicase protein interactions with other factors is extensive and beyond the scope of this review, but the reader can find some very useful review articles in this area [236,237,238,239,240,241,242,243].

## 8. Post-Translational Modifications of Helicase Proteins

Experimental studies over the years have provided strong evidence that the functions of DNA helicases, their interactions with protein partners, and their protein stability can be affected by specific post-translational modifications. Modulation of helicase function by post-translational modification provides a cellular mechanism for acute responses. The FANCJ helicase provides a good example. In 2003, Lee et al. reported that phosphorylation of FANCJ (then known as BRCA1 Interacting C-terminal Helicase or BACH1) mediates its specific interaction with the carboxyl-terminal domain of the tumor suppressor BRCA1 [244]. This interaction was determined to be required for DNA damage-induced checkpoint control. In other work, FANCJ was found to be acetylated at its extreme carboxyl terminus which enhances DNA end-processing that is a prerequisite for HR repair and normal checkpoint maintenance [245]. RecQ DNA helicases, including WRN [246] and BLM [247], are also subject to post-translational modifications which regulate their pathway functions in the DNA damage response and DNA repair. Helicase proteins modified by ubiquitylating ligases, acetyl transferases, or kinases can become susceptible to protein degradation, which may afford a more sustained response to changes in the cellular environment (for review, see [248]). New experimental approaches to assess the effects of post-translational modifications at the SM level [249,250] provide a more refined methodology for characterizing their outcome compared to earlier studies which dealt with mixed populations of helicase molecules. Assessing the impact of post-translational modifications of helicase proteins at high-resolution appears to still be at its infancy. This field is likely to expand rapidly and provide interesting new insights into the regulation of helicase activity.

## 9. Pathway Functions of DNA Helicases

In this section we provide a historical account of discoveries pertaining to DNA helicases and their pathway functions, which serves as a backdrop for the subsequent section on the roles of DNA helicases in genetic diseases and cancer. Due to its vast nature, we have elected to limit the discussion to the functional roles of DNA helicases in the replication stress response or regulation of gene expression. The roles of DNA helicases in DNA repair pathways are discussed in reference [251] and referred to in other sections of this review, so we will not address these here.

### 9.1. Replication Stress

To our knowledge, the *E. coli* RecQ helicase was the first DNA helicase implicated in a cellular response to environmental manipulation that causes replication stress. In 1984 Hanawalt and colleagues reported that a *recQ* mutant strain was resistant to thymineless death and displayed increased sensitivity to UV light [252]. Just over ten years later, the first discovery of a RecQ helicase disorder was reported [181] (see *Genetic Diseases* section, Section 10). As Hanawalt would discuss in a 2015 perspective [253], this early discovery of RecQ’s relationship to thymineless death provided a proof-of-principle that research in unicellular organisms can yield great insight into human health. RecQ was indeed found to be a DNA helicase and genetic evidence suggested its involvement in HR repair [67]. RecQ is also thought to function in conjunction with structure-specific nucleases (e.g., RecJ) at replication forks blocked by UV light-induced photoproducts [254]. As it turns out, physical and functional interactions of RecQ helicase with DNA nucleases acting at specific DNA junctions has been a prevailing theme over the years (see *Protein Interactions* and reference [76]). The helicase core domain of the *S. cerevisiae* Sgs1 helicase is highly conserved with *E. coli* RecQ [255]. A role for Sgs1 in resolving DNA topological stress was suggested by its interaction with a topoisomerase (e.g., Top3) [255]. A year after its discovery, Sgs1 was reported to interact with topoisomerase II, and this interaction was shown to be required for faithful chromosome segregation [256]. Sgs1 was also found to be important in suppressing cellular aging and in preventing nucleolar fragmentation [257]. Around this time (1997), it was discovered that mutation of *rqh1* (a Sgs1 homolog in *S. pombe*) compromised the ability of fission yeast to restart DNA synthesis after exposure to drugs that inhibit replication [258]. The Whitby laboratory showed that expression of a HJ resolvase in the *rqh1* mutant strain partially rescued sensitivity to UV light or hydroxyurea (HU), suggesting the aberrant accumulation of recombinational DNA intermediates when Rqh1 was absent [259]. Restarting stalled forks due to topological stress or DNA damage appears to be the primary role of Sgs1, and by analogy Rqh1. Loss of Sgs1 (or its human homolog BLM, defective in BS (see below)) leads to stalled fork structures acted upon by structure-specific nucleases. These events may underlie the elevated sister chromatid exchange (SCE) observed in BLM-deficient cells [260].

The role of BLM in genome stability is likely to be more complex than what is observed for RecQ homologs in lower eukaryotes. For example, BLM was found to associate with BRCA1 and other DNA repair proteins implicated in MMR (MSH2, MSH6, MLH1), double-strand break signaling/repair (ATM, RAD50-MRE11-NBS1), and replication (PCNA, RFC) in a multi-protein complex known as BRCA1-associated genome surveillance complex (BASC). This complex has been proposed to coordinate multiple DNA transactions during replication of DNA containing damage or unusually folded secondary structure [261]. BLM was also shown to associate with the FANCJ helicase in response to replication stress, and the two DNA helicases physically and functionally interact [262]. The Hickson laboratory reported that BLM is phosphorylated in response to cellular HU exposure, and the phosphorylated form of BLM is required for recovery from S-phase arrest [263]. It will be of interest to ascertain the mechanism whereby BLM acts to stabilize stalled replication forks or to promote recovery from S-phase arrest. It seems likely to be related to a role played by *S. cerevisiae* Sgs1, which has been shown by chromatin immunoprecipitation (ChIP) assays to be associated with the ATM-related kinase Mec1 that serves to stabilize the replicative DNA polymerases alpha and epsilon at forks stalled by HU [264,265].

Sgs1 is likely to operate in at least a partially redundant role with non-RecQ DNA helicases including SRS2 (already implicated in the intra-S phase DNA damage checkpoint) and the 5’ to 3’ DNA helicase RRM3, as shown by synthetic lethality analyses by microarray (SLAM), a novel experimental approach at the time [266]. Since then, many papers describing synthetic lethal interactions of DNA helicases have appeared in the literature suggesting a general theme that helicases respond to replication stress via extended networks with numerous genetic interactions (for examples, see [267,268,269,270]). The recent identification of WRN helicase (mutated in Werner syndrome (WS), see below) as a synthetic lethal gene in microsatellite unstable cancers with defects in DNA MMR genes emphasizes the point [271,272,273,274]. The challenge in functional genomics remains to understand how, and in what context, certain DNA helicases have unique pathway functions. For example, experimental evidence suggests that Sgs1 acts to prevent the accumulation of Rad51-dependent cruciform DNA structures at damaged replication forks that have undergone incomplete maturation of recombination intermediates [275]. Although there is only one RecQ helicase in yeast, there are five in humans implying unique functions for each. Indeed, distinct genetic disorders are attributed to mutations in three of the five human RecQ helicases (see *Genetic Diseases*, Section 10). Yet there seems to be overlap in DNA substrate specificity and protein partners. How tasks are divided among the human RecQ helicases in pathway functions during the replication stress response remains an active area of investigation.

While much of the focus on RecQ helicases and replication stress has historically centered on Sgs1 and BLM, the human WRN helicase-nuclease appears to also play an important role. This notion was first suggested by Pichierri et al. (2001) who determined that cells from WS patients displayed poor recovery from exposure to camptothecin or HU, leading to elevated strand breaks [276]. A technical breakthrough was made by the Monnat laboratory in 2007 when they showed that cellular recovery from replication fork arrest was compromised upon acute depletion of WRN by RNA interference [277]. Subsequently, Pirzio et al. presented experimental data suggesting a catalytic requirement for the WRN helicase activity, but not its exonuclease activity, in maintaining fragile site stability by suppressing chromosomal breakage at fork stalling sites [278]. A technique-driven advance in characterizing the role of WRN in the replication stress response was made when Sidorova et al. utilized DNA fiber analysis to show that WRN depletion reduced fork elongation rate in cells exposed to HU or the base alkylating agent methylmethanesulfonate [279]. Collapsed replication forks caused by ICL damage-induced double strand breaks are also resolved by WRN, as shown by the Bohr laboratory [280]. In this case WRN was proposed to facilitate ATM activation and S-phase checkpoint. This is in contrast to WRN’s role in response to mild replication stress in which WRN was observed to regulate ATR-driven checkpoint activation [281].

Cell-based SM studies from the laboratories of Alessandro Vindigni and Massimo Lopes have begun to address the apparently unique but coordinated roles of RECQL1, DNA2, and WRN to restart replication forks that have been stalled by various pharmacological agents. DNA topoisomerase inhibition causes replication forks to reverse, and RECQL1 helps forks restart by promoting fork reversal of regressed forks in a manner that is negatively regulated by Poly(ADP)ribose polymerase (PARP) 1 [103] (Figure 3D). WRN and DNA2, on the other hand, are involved in nucleolytic processing of reversed forks [104] (Figure 3E). DNA2 nuclease activity and WRN ATPase/helicase activity together degrade forks with a 5’ to 3’ polarity, which promotes replication restart. In our own work, RECQL1’s catalytic strand separation activity was demonstrated to facilitate normal fork dynamics by governing RPA’s availability during replication stress [282]. In the latest advance (2019), Sharma’s laboratory characterized a checkpoint-dependent role of RECQL1 to respond to replication stress induced by the chemotherapy drug gemcitabine [283].

In the last several years, Pavel Janscak’s group and their collaborators have led the charge to characterize RECQL5’s role in helping cells cope with replication stress. Their results suggest that RECQL5 helps to prevent fork stalling in genes actively being transcribed by RNA Polymerase I or II by alleviating collisions between transcription and replication machinery [284]. By another mechanism, RECQL5 is proposed to collaborate with MUS81 endonuclease to process stalled replication forks at common fragile sites [285]. In addition, RECQL5 was reported to disrupt RAD51 filaments on stalled forks at common fragile sites, making the transition from fork stalling at R-loops to replication restart go smoothly [286].

Although the RecQ helicases have dominated the landscape for study of the replication stress response, helicases of other families have also been implicated. The Fe-S cluster helicase FANCJ represents a good example. Beyond FANCJ’s role in ICL repair and HR repair of double-strand breaks, Gong et al. reported that FANCJ interacts with TopBP1, a protein known to be important for the DNA replication checkpoint that helps cells respond to stalled forks [287]. FANCJ’s interaction with TopBP1 was shown to be required for Chk1 and RPA phosphorylation after cellular exposure to a replication stress-inducing agent, which in turn is necessary for the replication checkpoint. It is plausible that the chromosomal instability observed in FANCJ-deficient cells [262] is at least partially attributed to a defective replication checkpoint. More recent work solidifies the importance of FANCJ in maintaining the genomic stability of microsatellite DNA sequences during replication stress [288,289]. We have determined, using biochemical and genetic approaches, that a minimal threshold of FANCJ catalytic activity is required to suppress chromosomal DNA damage during cellular conditions of replication stress [290]. The Cantor laboratory has reported that a delicate balance between FANCJ and the fork remodeling factor HLTF is necessary to maintain fork stability [105] (Figure 3F).

DNA helicases like *E. coli* RecG [106] or human WRN [107,108] and BLM [107,109] may promote fork regression when a replication fork stalls to allow fork protection and eventual replication restart (Figure 3G). Although not considered *bona fide* DNA helicases *per se*, ATP hydrolysis-driven DNA translocases share many features in common with DNA helicases and play a prominent role in the response to replication stress. The first to be described was HARP, now more commonly designated SMARCAL1 (SWI/SNF-related matrix-associated, actin-dependent regulator of chromatin, subfamily-like A1). SMARCAL1 interacts with RPA and aids in replication fork restart [291,292]. SMARCAL1 performs its cellular functions of fork regression and HJ branch-migration to maintain genomic stability when forks encounter DNA damage [293]. In addition to SMARCAL1, the DNA translocases ZRANB3 and HLTF are involved in remodeling stalled fork structures as well (see [294] for review).

### 9.2. Transcriptional Regulation

As components of the general transcription factor TFIIH, the XPB and XPD helicases help to regulate gene expression by RNA polymerase II [295]. Egly and Coin offered an excellent review on the history of TFIIH for readers to learn more about this protein complex and its regulatory roles [295]. In addition to transcriptional activation, both the XPB and XPD helicases contribute to NER [251,296]. Missense mutations in *XPB* or *XPD* are linked to genetic diseases (see below), and both helicase subunits are essential in humans and other eukaryotes. XPB ATPase activity is required to locally open the duplex region in the vicinity of the transcriptional start site, as well as enable promoter escape, early elongation, and re-initiation of transcription when RNA polymerase II stalls [295]. Although molecular evidence has demonstrated that the catalytic DNA unwinding function of XPD is not required for its role in TFIIH transcriptional activation, the absence of XPD altogether destabilizes TFIIH which severely compromises transcription [297].

Both XPB and XPD are thought to play an additional role in transcriptional regulation that is related to their interactions with G4 DNA structures. G4 motifs are known to be enriched in promoter regions [298], suggesting that G-quadruplex structures residing within or downstream of promoters are utilized by the cell for transcriptional regulation. The Maizels laboratory pursued the hypothesis that the XPB and XPD helicases bind genomic G4 DNA structures, and the protein: DNA interactions are important for their transcriptional regulatory roles [98]. By ChIP Sequence analysis, they were able to show that 40% of the XPB and XPD binding sites overlapped with human DNA sequences predicted to form G4. Although a direct linkage of XPB or XPD G4 binding to transcriptional regulation was not shown, the authors determined that the *S. acidocaldarius* XPD helicase resolves a G4 DNA substrate in vitro. Further studies in this area are necessary for a mechanistic understanding of the importance of XPB or XPD interaction with promoter-localized G4 DNA in transcriptional regulation.

Of the five RecQ helicases, the strongest evidence for a primary role in regulation of RNA polymerase II transcription exists for RECQL5. The first report of RECQL5’s interaction with RNA polymerase II was in 2008, when its role in negatively regulating transcription was proposed [299]. RECQL5 was subsequently shown to suppress double strand breaks dependent on RNA polymerase II transcription [300]. In reconstitution experiments, RECQL5 was shown to inhibit both initiation and elongation by RNA polymerase II [301]. Domains in RECQL5 that interact with the initiation and elongation forms of RNA polymerase II were mapped [302]. Conversely, RECQL5 was shown to interact with a phosphorylated C-terminal domain of RNA polymerase II during transcription elongation [303]. Structural studies showed that RECQL5 binds to a site that overlaps with the binding site for the transcription factor TFIIS, positioning itself such that transcription elongation is sterically blocked [304]. An analysis of the genome-wide RNA polymerase II density profile suggested that RECQL5 acts to regulate movement of RNA polymerase II across genes in a manner that reflects transcription stress, where chromosomal breakpoints are localized [305]. An understanding of the basis for RECQL5’s modulatory effect on RNA polymerase II transcription began with the finding that the cellular topoisomerase TOPI is SUMO-lyated in a RECQL5-dependent manner, which prevents it from causing DNA damage at transcriptionally active chromatin [306]. Based on further studies in human cells, a model was proposed that RECQL5 helicase helps to prevent replication-transcription collisions [284]. A role for RECQL5 in restarting DNA replication after encounters with co-transcriptional R-loops and in collaboration with numerous other DNA repair factors was proposed recently [286].

Evidence suggests that G4-resolving or G4-interacting RecQ helicases influence gene expression. Brad Johnson’s laboratory provided the first evidence that cellular deficiency of the G4-resolving WRN and BLM helicases perturbs expression profiles of genes with G4-forming potential, as demonstrated by microarray analyses [307]. Further studies suggested that a deficiency in WRN [308] versus BLM [309] resulted in differential regulation of distinct sets of genes with enriched G4 motifs at the transcriptional start site or exon 1/intron 1 boundary, suggesting unique roles of the two helicases in gene expression. ChIP experiments suggested that RECQL1 preferentially binds to G4-forming genomic DNA sequences in a manner that is important for regulation of gene expression as well [310]. There is much yet to be learned regarding the role of DNA helicases in transcriptional regulation.

## 10. Genetic Diseases Characterized by Premature Aging and Cancer Linked to Molecular Defects in DNA Helicases

The decade of the 1990s marked an important era in helicase discovery as it relates to human disease. It was during this period that hereditary disorders characterized by DNA repair defects and/or chromosomal instability, a predisposition to cancer, and in certain cases, accelerated aging phenotypes were linked to mutations in helicase genes. Discovery of new rare hereditary disorders characterized by features of accelerated aging and/or cancer continues in the 21st Century. With a 2011 estimate of 31 nonredundant DNA helicases encoded by the human genome [14], it seems probable that more DNA helicase-linked genetic diseases will be identified, given the fundamentally important roles of ATP-dependent DNA unwinding enzymes in nucleic acid metabolism. However, as suggested in a recent review by Ray Monnat and colleagues, loss of function or compound haploinsufficiency for certain DNA helicase genes (e.g., *RECQL1, RECQL5*) may not yet be linked to a disease because their absence or dysfunction is incompatible with life (i.e., embryonic lethal) [311] or the rare causative mutation has not been identified. Genomics is still a young field and there are individuals worldwide with rare diseases in which the causative mutations have not yet been precisely mapped as challenges in the application of genome sequencing still exist [312]. In the following sections, we will describe several well-known genetic disorders attributed to mutations in DNA helicase genes from a historical perspective so the reader can appreciate the steady progress being made.

### 10.1. Xeroderma Pigmentosum, Cockayne Syndrome, and Trichothiodystrophy

In 1990, a helicase gene mutated in the skin cancer disease Xeroderma pigmentosum was identified by cDNA cloning and genetic complementation of the UV light sensitivity defect in UV5 Chinese hamster ovary cells [313]. This gene, originally designated *ERCC2* and later dubbed *XPD*, was observed to share sequence similarity with the yeast *RAD3* gene implicated in NER. Interestingly, XPD has roles in both DNA repair and transcription. Moreover, molecular defects in XPD are responsible for three distinct genetic disorders: Xeroderma pigmentosum, Xeroderma pigmentosum combined with Cockayne syndrome, and Trichothiodystrophy [314]. The purified *XPD* gene product was confirmed to be a DNA-dependent ATPase and DNA helicase in subsequent work [315]. XPD’s ATPase/helicase function are required for NER but not its role in transcription [297]. Based on collective evidence from the laboratories of Naegeli [316] and Hanaoka [317], it seems likely that XPD’s role in NER damage recognition is to scan the DNA molecule to verify the lesion (for review, see [318]).

For a comprehensive review of the multi-faceted roles of XPD in cellular nucleic acid metabolism, please see [319]. By virtue of its Fe-S cluster, XPD is capable of DNA-mediated redox signaling [320]. The Barton laboratory proposed a model in which DNA charge transport allows XPD and other redox active DNA repair proteins to efficiently search for DNA damage in the genome [321]. As discussed below, other human DNA helicase genes sharing sequence similarity in the helicase core domain with XPD are also implicated in hereditary disorders characterized by chromosomal instability and DNA repair defects.

Mutations in a second gene encoding a putative DNA helicase (originally designated *ERCC3* and subsequently named *XPB*) were linked to Xeroderma pigmentosum and Cockayne syndrome in 1990 [322,323]. XPB mutations are also implicated in Xeroderma pigmentosum with neurological abnormalities and Trichothiodystrophy [324]. As mentioned above, XPB (like XPD) is involved in both NER and transcription.

### 10.2. Bloom Syndrome

For many years it was known that the hypermutability associated with BS was accompanied by replication defects and elevated SCE, the latter serving as a clinical diagnostic test for the disorder [325]. In 1995, Ellis, German and colleagues utilized a classical mapping technique known as positional cloning to determine the location and identity of bi-allelic BS-causing mutations in a novel gene (designated *BLM*) sharing sequence similarity with the coding region for the ATPase/helicase core domain of the bacterial RecQ helicase [181]. This discovery was not only exciting for scientists interested in aging, but also those studying cancer biology. Individuals with BS display a broad spectrum of blood and solid-tumor cancers as well as immuno-deficiency, short stature, and certain features of rapid aging. The apparently contrasting roles of the BLM helicase to either promote or dissuade recombination has made it difficult to grasp the molecular basis for the elevated SCE observed in BS. However, biochemical studies from the Hickson laboratory using oligonucleotide-based or plasmid-based double HJ substrates suggests that BLM has a unique role among the RecQ helicases. BLM collaborates with a DNA topoisomerase to dissolve the complex structure which is a presumed intermediate of HR or converging replication forks [326].

In addition to its role in modulating recombinational DNA repair by branch-migrating mobile displacement (D)-loop substrates [327] or acting upon HJ structures [63], experimental evidence suggests that BLM plays a role in DNA end-resection, a process that is required to generate a single-strand overhang for strand invasion into homologous duplex DNA. From in vitro reconstitution experiments, BLM was shown to act in complex with the structure-specific helicase-nuclease DNA2 or EXO1 to perform long strand resection [328,329]. BLM has also been implicated in protection and repair of replication forks [330] and resolution of ultra-fine anaphase DNA bridges [331]. However, it is still unclear how defects in these BLM-related activities are responsible for the cellular and clinical features of BS. Presumably, BLM helicase has multiple roles in DNA transactions in vivo and its molecular deficiency contributes to chromosomal instability in BS through several avenues [332].

### 10.3. Werner Syndrome

The discovery of the *BLM* gene was rapidly followed in the subsequent year (1996) with a report by Yu et al. detailing the positional cloning of the gene mutated in the premature aging disorder WS [333]. The *WRN* gene, defective in WS, also encodes a DNA helicase of the RecQ family. Interestingly, a conserved exonuclease domain, characteristic of proofreading nucleases, was also identified in WRN [334] making it unique among the five human RecQ helicases. WS is distinct from BS in that it much more dramatically displays many of the clinical features of aging early in life. The clinical features appear after adolescence, typically in the mid to late 20’s, in an affected person. A particularly remarkable aspect of WS is that practically all of the symptoms of normal aging, including diabetes, osteoporosis, cataracts, heart disease, wrinkled skin and gray hair, manifest early in life and the average lifespan is 46 years. Neurological decline is generally not thought to be a symptom of WS. However, due to the premature death of WS patients and the limited number of cases in the WS registry, the prevalence of clinical features resembling early Alzheimer’s Disease or dementia in some WS patients cannot be excluded. See [335] for a recent discussion of WS and the central nervous system. A clinical feature that distinguishes WS from BS is that cancer types in WS are less broad than in BS. Soft tissue sarcomas and other neoplasms are prevalent in WS, whereas BS displays a broad spectrum of cancers including solid tumor and blood cancers [336].

From a historical perspective, a significant advance in understanding the molecular pathology of WS was made by discoveries linking WRN defects to abnormal telomere metabolism. This began with mouse model studies showing that co-deficiency of WRN and the telomerase RNA-protein complex in later generation mice with short telomeres displayed accelerated aging and chromosomal instability as is typically observed in human WS patients [337,338,339]. Furthermore, human cells defective in WRN helicase activity were found to display defective lagging strand synthesis in the telomeres [340]. Following these advances, biochemical characterization of the WRN protein revealed a DNA substrate specificity and protein interactions that suggested a specialized molecular role in telomere metabolism [234]. More general roles of WRN in replication and recombination are also likely to come into play. Despite the work of many laboratories and over two decades of research, the molecular basis for WS remains enigmatic. Perhaps novel approaches that examine the crosstalk of DNA damage with cellular metabolism and senescence observed in other forms of progeria (e.g., [341]) and a focus on why WS is an adult-onset disorder [342] may help to move the field forward.

### 10.4. Rothmund-Thomson Syndrome and Related RECQL4 Disorders

In 1999 Rothmund-Thomson syndrome (RTS) was shown to be linked to bi-allelic mutations in a third member of the RecQ family, the RECQL4 helicase [343]. Further studies revealed that RECQL4 mutations are linked to not only RTS, but also Baller-Gerold syndrome [344] and RAPADILINO syndrome [345], all characterized by stunted growth, radial ray defects, and skeletal abnormalities. The molecular basis for the complex disease symptoms associated with RECQL4 mutations remains to be determined, but the recent structure of RECQL4 may help to explain the defects caused by some of the mutations [198]. Of the five human RecQ helicases, RECQL4 appears to have the weakest ATP-dependent DNA unwinding function in vitro [346,347]. Like the other RecQ helicases it can also perform strand annealing [348], the opposite of DNA unwinding. Adding to the complexity, recent work using mouse models suggests that RECQL4 helicase activity is not required for normal development or physiological functions [349]. Consequently, it remains unclear if defective RECQL4 helicase activity in humans *per se* is implicated in any of the three RECQL4-linked genetic disorders. Consistent with this hypothesis, the essential role of RECQL4 in hematopoiesis, as shown in RECQL4-deficient mice, was found not to be dependent on its helicase activity [350], leaving the importance or role of its modest DNA unwinding activity unexplained.

### 10.5. Fanconi Anemia

As alluded to above, a new cadre of clinically relevant diseases have been identified in which the mutated genes all encode proteins which share sequence similarity within the helicase core domain of XPD. Remarkably, in 2005 three research teams independently discovered that mutations in a DNA helicase gene (designated *FANCJ* and sharing sequence similarity with XPD) were implicated in a genetically complex disorder known as FA characterized by congenital abnormalities, progressive bone marrow failure, and cancer [351,352,353]. A peculiar feature of the XPD/FANCJ helicase family is the presence of an Fe-S cluster [354] that serves a critical structural and biochemical function most likely as a molecular wedge in helicase-catalyzed duplex DNA unwinding [192,193,354]. Consistent with these findings, a pathogenic amino acid substitution in the Fe-S domain of FANCJ was shown to abolish its DNA repair function in vivo and inactivate its helicase function in vitro [355]. Cells from individuals carrying bi-allelic FANCJ mutations, like the mutations in other FA genes, are hypersensitive to DNA cross-linking agents. It is believed that FANCJ’s role in HR repair is critical for healing of double-strand breaks associated with interstrand cross-linking (ICL)-induced DNA damage in replicating cells (for review, see [356]). However, FANCJ’s molecular mechanism of action remains poorly understood. In addition, FANCJ plays an even broader role in the cellular response to replication stress [105,290]. It was recently shown by biochemical and genetic studies that a greater threshold of FANCJ helicase activity is required for a robust response to ICL-induced damage compared to double-strand breaks induced by the chemical bleomycin or replication stress caused by the DNA polymerase inhibitor aphidicolin or G-quadruplex ligand telomestatin [290]. With emerging evidence that endogenous macromolecule damage induced by formaldehyde and aldehyde derivatives may be the causative force underlying FA [357,358], a central challenge will be to determine how molecular defects in FANCJ and the 20+ other proteins implicated in the FA pathway are responsible for the characteristic disease outcomes, i.e., accelerated decline of the hematopoietic stem cell compartment and other features of aging [359].

In addition to FANCJ, a second DNA-dependent ATPase designated FANCM was discovered and bi-allelic mutations were originally thought to be linked to FA [360]. However, this turned out not to be the case as that individual harbored bi-allelic mutations in the *FANCA* gene [361], which was already shown to be genetically linked to FA [362]. More recent work implicates FANCM as a facilitator of HR and replication by collaborating with a number of genome caretaker proteins. Consistent with this proposal, FANCM is mutated in various cancers and suspected to act globally as a tumor suppressor (for review, see [363]). From a biochemical standpoint, FANCM was shown in 2005 by Meetai et al. to possess a dsDNA translocase activity. However, FANCM is not a *bona fide* DNA helicase capable of catalytically separating complementary strands of a duplex DNA substrate [360]. Further studies revealed that FANCM is capable of branch-migrating HJs and certain DNA structures associated with stalled replication forks [364,365,366,367]. FANCM’s branch-migration activity may be relevant to its collaborative role with the BLM helicase to suppress SCEs and confer resistance to DNA cross-linking agents [368]. Using an elegant SM technique to visualize replication forks as they encounter ICLs, Seidman’s laboratory provided definitive evidence that FANCM’s ATPase/translocase activity facilitates replication traverse of the ICL [369]. The molecular mechanism(s) of ICL tolerance and repair continues to advance rapidly with novel experimental strategies and model systems; the reader is referred to several recent reviews on this topic [370,371,372,373].

### 10.6. Dyskeratosis Congenita and Warsaw Breakage Syndrome

The RTEL1 helicase, mutated in Dyskeratosis congenita (DC) or its more severe form Hoyeraal-Hreidarsson syndrome (HHS), and the DDX11 helicase mutated in Warsaw Breakage syndrome (WBS) have most recently joined the group of clinically relevant Fe-S DNA helicases. Both diseases are chromosome instability disorders, but unique in their presentation. Cells from WBS individuals display elevated sister chromatid cohesion defects [374], whereas DC or HHS, due to RTEL1 mutation, is characterized by telomere shortening [375,376,377]. Evidence suggests that RTEL1 plays a role in the processing of telomeric D-loops (so-called t-loops), resolution of telomere-associated G-quadruplex DNA [378], and regulation of HR repair [379]. Until only very recently, definitive molecular evidence for RTEL1’s precise role(s) was lacking. However, new studies by Sarek et al. demonstrate that RTEL1’s access to t-loops to enable smooth telomere replication during the cell cycle is governed by the phosphorylation state of the shelterin protein TRF2 [380]. A more general role for RTEL1, acting together with PCNA in replication of the whole genome, has also been suggested based on DNA fiber studies of mouse embryonic fibroblasts from RTEL1 mutant mice [123].

Interest in the biology of DDX11 helicase continues to grow with the identification of new patients [381], development of cell-based models [382,383,384,385], and an increased understanding of the molecular events important for sister chromatid cohesion [386,387,388]. However, compared to the Fe-S helicases XPD, FANCJ, and the human RecQ helicases, the mechanism and functional importance of helicase-catalyzed DNA unwinding by DDX11 (or RTEL1) has been largely under-studied. Research from the Pisani laboratory suggests that the interaction of DDX11 with the replication fork-protection factor Timeless is important for fork recovery from replication stress and promotion of sister chromatid cohesion [383,384]. A role of Timeless as a checkpoint protein that couples cell cycle progression with circadian rhythm [389,390] raises the question if DDX11 or other DNA helicases implicated in the DNA damage response and genome maintenance are involved in the cell-autonomous clock that regulates physiologic functions.

### 10.7. Mitochondrial Twinkle Helicase Diseases

Hereditary mitochondrial diseases attributed to mutations in the human nuclear-encoded mitochondrial replicative helicase Twinkle (*C10orf2*) include progressive external ophthalmoplegia (PEO), ataxia-neuropathy syndromes (e.g., infantile-onset spinocerebellar ataxia), and mitochondrial DNA depletion syndromes (e.g., hepatocerebral syndrome) (for review, see [391,392]). The genetics of Twinkle-linked mitochondrial disorders is complex, and often the inheritance is attributed to an autosomal dominant mutation. The Copeland, Falkenberg, and Spelbrink laboratories have undertaken efforts to characterize a number of clinically relevant Twinkle mutations and their biochemical effects, which suggest partial reduction in activity, destabilization of the protein, and interference in oligomerization [393,394,395]. The *Twinkle* gene was first discovered in 2001 by Spelbrink et al. [396]. In this work, they identified Twinkle mutations linked to adult onset PEO characterized by multiple mitochondrial deletions. A number of groups have been involved in the biochemical and biophysical characterization of Twinkle which, like other replicative helicases, forms multi-subunit (6 or 7-membered) rings [397,398]. A transgenic mouse model for a Twinkle mutation was found to recapitulate the PEO characteristic of the human disorder [399].

## 11. Helicase Mutations and Predisposition to Cancer

Although the genetic linkage of DNA helicase defects to rare chromosomal instability disorders has been emphasized in this section, it should be stated that there are numerous studies documenting mutations in DNA helicase genes as being associated with cancers [400]. For example, a recent study established that rare missense alleles of BRIP1/FANCJ confer risk for breast as well as ovarian cancer [401]. This work is consistent with a previous study that truncating mutations in BRIP1/FANCJ confer susceptibility to breast cancer [402]. The association of helicase mutations with various cancers, as well as hereditary diseases with a cancer predisposition, is believed to reflect the vast and important roles of DNA helicases in DNA repair, maintenance of genomic stability, and cellular checkpoint responses important for chromosomal stability. Thus, DNA helicases belong to a class of genome caretaker proteins important for cancer suppression.

## 12. Small Molecule Modulation of DNA Helicases

We have discussed how helicase function can be modulated by mutation, post-translational modification, or protein interaction. Yet another form of helicase regulation can be achieved by pharmacological modulation with helicase-interacting small molecules. This field of study was motivated in large part by earlier reports that DNA repair proteins (e.g., (PARP)) are inhibited by specific small molecules (e.g., olaparib in the case of PARP-1) which can be used to modulate DNA repair capacity in human cells, particularly in defined genetic backgrounds (BRCA) [403,404]. Such an approach is postulated to be a new methodology for anti-cancer therapy, and PARP inhibitors are being used in the clinic (e.g., olaparib [405], rucaparib [406]) or in clinical trials. Other DNA repair inhibitors are currently being investigated for their vulnerability to pharmacological inhibition by small molecules that interact with their targets [407].

The first reported class of helicase inhibitors were targeted against viral helicases in an effort to suppress viral diseases, dating back to the early 2000’s (for review, see [408]). The first described human helicase inhibitor was directed against WRN [409], followed by a BLM helicase inhibitor [410]. These compounds were found to be bioactive with human cells grown in culture in a helicase target-dependent manner, suggesting their specificity. A second WRN helicase inhibitor, structurally related to the first, was found to be effective in sensitizing FA mutant cells to very low concentrations of an ICL-inducing agent [411], suggesting that the WRN-catalyzed DNA unwinding function helps cells cope with ICL-induced DNA damage possibly by a replication stress response or an HR-dependent mechanism. More recently discovered small molecule inhibitors directed against helicase proteins such as the human DNA2 helicase-nuclease [412,413], human immunodeficiency virus (HIV) DDX3 RNA helicase [414,415], and the eukaryotic MCM replicative helicase [416,417,418] have been described. Important advances for the development of helicase inhibitors are likely to come with organismal genetic models (e.g., mice) and successful virtual screening to develop more potent and specific helicase inhibitors [419]. Although still far from clinical application, small molecule helicase inhibitors have already proven to be useful tools in basic science research.

## 13. Summary

In this review we have provided a historical perspective of the study of DNA helicases by highlighting some of the seminal discoveries for this unique class of nucleic acid metabolizing enzymes. Our chronological account is meant to recognize key findings that have helped to shape this fertile area of study and provide a resource for early career nucleic acid biologists. In addition, we hope that this review will inspire researchers to continue their pursuit of new directions for investigation of DNA helicases in the years to come. While RNA helicases were not discussed in this review, we recognize that these enzymes are equally tantalizing in their interest and impact.

## Figures and Tables

**Table 1:** Helicases that Resolve Triplex and G-Quadruplex DNA Structures ^a^.

Organism/Virus	Resolving Helicase	Reference
Triplex DNA		
SV40	large T antigen	[110]
*H. sapiens*	WRN	[111]
*H. sapiens*	BLM	[111]
*E. coli*	RecQ ^b^	[112]
*H. sapiens*	DHX9	[113,114]
*H. sapiens*	DDX11 (ChlR1)	[115]
*S. cerevisiae*	XPB ^c^	[116]
G-quadruplex DNA		
*E. coli*	RecQ	[117]
*H. sapiens*	BLM	[65,118]
*S. cerevisiae*	Sgs1	[119]
*H. sapiens*	WRN	[65,120]
*H. sapiens*	FANCJ	[82,83,121]
*S. cerevisiae*	Pif1	[91]
*H. sapiens*	Pif1	[92,122]
*H. sapiens*	RTEL1	[123]
*H. sapiens*	DDX11 (ChlR1)	[124]
*S. acidocaldarius*	XPD	[98]

^a^ The list is meant to be representative, but not exhaustive, of DNA helicases demonstrated to resolve G4 structures in vitro. ^b.^ Triplex resolving activity inferred based on primer extension assays with triplex DNA substrate; see reference for detail. ^c.^ TFIIH with ATPase-defective Rad3 was purified from yeast and tested in the assay.

**Table 2:** Selected Prominent DNA Helicase Structures ^a^.

Year	Helicase ^b^	Significant Finding ^c^	Reference
1989	SV40 Large T antigen	Double hexamer formed on dsDNA in presence of ATP	[173]
1991	*E. coli* rho	Hexamer ring stabilized by RNA ^d^	[183]
1994	*E. coli* RuvB	Dodecamer of double hexameric rings around dsDNA	[184]
1995	T7 gene 4	Hexameric ring-like structure around ssDNA	[174]
1996	*B. stearothermophilus* PcrA	First helicase crystal structure; two RecA-like domains with cleft for nucleotide binding	[50]
1997	*E. coli* Rep	First reported crystal structure of helicase bound to DNA	[52]
1999	*T. thermophilus* UvrB	Helicase core, DNA and ATP binding domains revealed	[185]
1999	*B. caldotenax* UvrB	Structure used to predict UvrB pre-incision complex	[186]
1999	*B. stearothermophilus* PcrA	Monomer bound to DNA suggesting an inchworm mechanism	[51]
1999	T7 gene 4	Hexamer subunit interactions and nucleotide binding characterized	[187]
2000	T7 gene 4	DNA translocation mechanism by sequential nucleotide hydrolysis	[188]
2003	*E. coli* RecQ	Conserved Zn^2+^-binding and winged helix domains in RecQ helicases	[179]
2004	*E. coli* RecBCD	Coupling mechanism for motor activities and nuclease shown	[189]
2006	Papillomavirus E1	ssDNA passes through ring; escort mechanism for DNA translocation	[190]
2006	*E. coli* UvrD	Two-part power stroke (1 bp unwound per ATP hydrolyzed)	[131]
2007	*B. stearothermophilus* DnaB	Two-layered ring of hexameric DnaB bound to DnaG Primase	[175]
2008	*S. tokodaii* XPD	Two RecA-like domains, Fe-S cluster, and Arch domain revealed	[191]
2008	*S. acidocaldarius* XPD	Conserved RecA, Fe-S cluster and Arch domains identified	[192]
2008	*T. acidophilum* XPD	Fe-S domain contributes to donut shape for strand separation	[193]
2008	*S. solfataricus* MCM	6-fold symmetry of hexamer; nucleotide-binding pocket at interface	[176]
2009	*D. radiodurans* RecD2	Nucleotide-induced conformational changes; basis for DNA translocation directionality	[53]
2009	*H. sapiens* RECQ1	Winged helix domain harbors β-hairpin strand-separation pin	[194]
2012	*T. acidophilum* XPD	Basis for uni-directional ssDNA translocation polarity (5’ -3’)	[54]
2015	*S. cerevisiae* Mcm2-7	Cryo-EM structure suggests narrow passageway fitting duplex DNA	[177]
2015	*H. sapiens* BLM	Conformational role of HRDC domain; base-flip unwinding proposed	[195]
2016	*H. sapiens* RECQ1	Strand separation pin buttressed by protein dimer interface	[196]
2016	*S. cerevisiae* CMG ^e^	Inchworm mechanism with pumpjack motion for translocation	[197]
2017	*H. sapiens* RECQL4	Novel C-terminal domain not found in other human RecQ helicases	[198]
2017	*H. sapiens* RECQL5	Nucleotide binding dependent open and closed conformations	[199]
2018	*E. coli* DinG	Mechanism for unidirectional translocation and lesion stalling	[200]
2018	*S. cerevisiae* Pif1	G4-stabilized dimer formation and a potential G4 binding site	[201]
2018	*C. sakazakii* RecQ	RecQ bound to resolved G4; guanine-flipping and sequestration	[202]
2019	*Bacteroides* sp. Pif1	Coordination of two Pif1 molecules for unwinding forked DNA	[203]
2019	*S. solfataricus* MCM	DNA translocation mechanism of MCM complex characterized	[172]

^a^ Chronological list is meant to be representative, but not exhaustive. ^b^ Presence of nucleotide and/or DNA is not indicated; see reference for detail. ^c^ Major discovery listed; see reference for details of reported findings. ^d^ Note: presence of RNA, not DNA. ^e^ CMG; Cdc45-Mcm2-7-GINS.
